# The Central Hospitals Board

**Published:** 1896-05-16

**Authors:** Sydney Holland


### The Institutional Workshop.

### THE CENTRAL HOSPITALS BOARD.

By the Hon. Sydney Holland.

I recognise in Dr. Allchin a foe worthy of one's steel—a man whose position, work, experience, and, if I may add, character entitle his opinion to every possible respect. I have been attacked elsewhere by "Hospital Secretary" and "Philanthropist"; but men who attack me under a *nom de plume* cannot expect, and probably do not want, a reply. It is tempting to reply to them, because these anonymous scribes are so virulent and delightfully personal. I wish, before replying to Dr. Allchin, to make one introductory remark, and it is this: Because I criticise others I do not pretend to be better myself. That is an absolutely false position to take against me. I entirely disagree with the proverb that those who live in glass houses should not throw stones. They are the very people who should, because they know better than anyone else the effects of stone-throwing. So let me pose, please, as an average sinner trying to improve. What attracts me to the Central Board principle is that I have really tried to approach the great work of hospital management by gaining all I can from the experiences of older and wiser men, and from other hospitals than the one with which I happen to be immediately connected, and of which, I am glad to say, Dr. Allchin has done us the honour of accepting the post of consulting physician. But I have found that there is no focussed experience outside the pages of the "Hospital Annual," there is no centre of information, all the hospitals are at sixes and sevens on questions of vital importance, they overlap each other's work, there is no inter-communication of any sort—except the Hospital Officers' Club—and if we want information and advice it must be got at the expense of much time and trouble in going round all the hospitals, or in lengthy correspondence.

Dr. Allchin puts the arguments against a Central Board with, as might be expected of him, fairness and force. But the weak spot in his armour is that he looks on the question as an insider and not at all as an outsider, and I think he overlooks the point that a refusal on the part of the hospitals to meet broadly and generously the cry for the better organisation of the enormous amount of free medical relief now given in London, is a disturbing element in that confidence of the public in their management without which hospitals cannot continue their work. Apart from the matters I have referred to above, I do not think Dr. Allchin could seriously deny that some concerted action is needed on the part of all hospitals to lessen the damage done by them to the local practitioners.

Some minds are so constituted that they foresee danger in every proposed change. My worthy great-grandfather, Sydney Smith (I may boast, I hope, without being thought conceited, of being descended from so great a lover of his fellow-men, especially as he himself claimed no great heritage of birth; "My ancestors disappeared about the time of the summer assize, and were never heard of again," he used to say), declared on his oath that if St. Paul's Cathedral were opened to the public, nuisances of every sort, which I need not particularise, would be committed in it, and the Cathedral would be the resort of every evil doer. But he was wrong. Dr. Allchin may have a similar mind, and possible evils may weigh with him more than certain good. I write "certain good" because no one will dispute that, if properly constituted and if properly managed, a central board would do good.

Let me take the most popular of Dr. Allchin's arguments—that for a Central Board to report on every hospital is to hand hospitals over to the terrorism of the Press, which dearly loves a hospital scandal—that the imperfect information based on the one side of the question put forth by the Central Board would be no bar to the avidity of the Press. Certainly there is some truth in this, but it is what I may call a wholesome fear, I mean a fear out of which good might and would come and has come in the case of Dublin hospitals, where the Hospital Sunday Fund each year does issue such reports. This is the fear which has prevented many a man from doing what he thought right. It is the old "What will people say?" instead of "Do right and shame the devil" (which includes the printer's devil). I insisted, however, that the hospital should be heard in its defence, and that defence would be given the same publicity. The fear would be felt more by the evil-doers than by the good. Surely, too, it is not expecting too much of the committee, composed of, as I suggested, five laymen, five hospital, four medical, and one nursing representative, and all these picked men from a larger representative body, that they would exercise the greatest care before putting into any report of theirs a statement likely to damage a hospital. This is just an instance of a fear which it is impossible to combat by argument, and the magnitude of it, or the contempt for it, depends on the disposition of each individual man. I am sure of one thing, and that is that I myself, and the hospital I am connected with, have been much more severely handled by the Press than Dr. Allchin or the Westminster have ever been. And the worst of it was that we deserved it. I wish to be courteous to Dr. Allchin and not to seem to despise his fears, but if anyone will re-read his letter it will be seen that there is no single argument brought forward against the Central Board principle, but only a repetition of evil prophecy and vague fear, which the experience of Dublin does not confirm.

Dr. Allchin writes that the "Committee of the House of Lords numbered amongst its members some of the ablest men." Admitting this, is it not likely they were right in unanimously recommending that a Central Board should be formed, and that Dr. Allchin is wrong in stigmatising such a recommendation as "impracticable and mischievous"? True, they heard but very little evidence on the subject. But they had sat for weeks, and had learned the chaos of customs and rules existing at the different hospitals; they had gathered what was needed, and ventured to recommend a remedy founded on their business and general experience. Their recommendation has been generally accepted by really almost every thoughtful man outside hospital managers, and I can assure Dr. Allchin sincerely that I did not make the statement lightly, that the principle of a Central Board was admitted by a large number of those who, like myself, are engaged in hospital management, and that it was wished for by many of Dr. Allchin's honourable profession. Dr. Allchin may remember that even the mover of the amendment at the Charing Cross meeting declared himself in favour of a Central Board. So there is no question of hospitals going back on a policy. I am, however, very anxious that we hospital workers should not wait till a Central Board is perhaps forced upon us by outside opinion and outside people.

